# Afucosylated IgG Targets FcγRIV for Enhanced Tumor Therapy in Mice

**DOI:** 10.3390/cancers13102372

**Published:** 2021-05-14

**Authors:** Rens Braster, Marijn Bögels, Hreinn Benonisson, Manfred Wuhrer, Rosina Plomp, Arthur E. H. Bentlage, Rianne Korthouwer, Remco Visser, J. Sjef Verbeek, Marjolein van Egmond, Gestur Vidarsson

**Affiliations:** 1 Department of Molecular Cell Biology and Immunology, VU University Medical Centre, 1081 HZ Amsterdam, The Netherlands; rsb@genmab.com (R.B.); mbogels@cmumed.org (M.B.); Rianne.korthouwer@Inholland.nl (R.K.); m.vanegmond@amsterdamumc.nl (M.v.E.); 2 Department of Human Genetics, Leiden University Medical Centre, 2333 ZA Leiden, The Netherlands; hreinn.benonisson@med.lu.se (H.B.); j.s.verbeek@toin.ac.jp (J.S.V.); 3 Center for Proteomics and Metabolomics, Leiden University Medical Center, 2333 ZA Leiden, The Netherlands; m.wuhrer@lumc.nl (M.W.); rosinaplomp@hotmail.com (R.P.); 4 Sanquin Research and Landsteiner Laboratory, Academic Medical Centre, Department of Experimental Immunohematology, University of Amsterdam, 1066 CX Amsterdam, The Netherlands; A.Bentlage@Sanquin.nl (A.E.H.B.); R.Visser@sanquin.nl (R.V.); 5 Department of Surgery, VU University Medical Centre, 1081 HV Amsterdam, The Netherlands

**Keywords:** melanoma, afucosylated IgG, Fc-receptors

## Abstract

**Simple Summary:**

Cancer treatments are increasingly based on therapeutic antibodies to clear tumors. While in vivo mouse models are useful to predict effectiveness of human antibodies it is not completely clear how useful these models are to test antibodies engineered with enhanced effector functions designed for humans. One of the changes considered for many new antibody-based drugs is the removal of fucose (resulting in afucosylated IgG) which enhances IgG-Fc receptor (FcγR) mediated effector functions in humans through FcγRIIIa. Here we show that afucosylated human IgG1 also have enhanced effector functions against peritoneal metastasis of melanoma cells in mice through the evolutionary related mouse FcγRIV. This shows that afucosylated human IgG is functionally recognized across species and shows that mouse tumor models can be used to assess the therapeutic potential of afucosylated IgG1.

**Abstract:**

Promising strategies for maximizing IgG effector functions rely on the introduction of natural and non-immunogenic modifications. The Fc domain of IgG antibodies contains an N-linked oligosaccharide at position 297. Human IgG antibodies lacking the core fucose in this glycan have enhanced binding to human (FcγR) IIIa/b, resulting in enhanced antibody dependent cell cytotoxicity and phagocytosis through these receptors. However, it is not yet clear if glycan-enhancing modifications of human IgG translate into more effective treatment in mouse models. We generated humanized hIgG1-TA99 antibodies with and without core-fucose. C57Bl/6 mice that were injected intraperitoneally with B16F10-gp75 mouse melanoma developed significantly less metastasis outgrowth after treatment with afucosylated hIgG1-TA99 compared to mice treated with wildtype hhIgG1-TA99. Afucosylated human IgG1 showed stronger interaction with the murine FcγRIV, the mouse orthologue of human FcγRIIIa, indicating that this glycan change is functionally conserved between the species. In agreement with this, no significant differences were observed in tumor outgrowth in FcγRIV^-/-^ mice treated with human hIgG1-TA99 with or without the core fucose. These results confirm the potential of using afucosylated therapeutic IgG to increase their efficacy. Moreover, we show that afucosylated human IgG1 antibodies act across species, supporting that mouse models can be suitable to test afucosylated antibodies.

## 1. Introduction

The use of monoclonal antibodies (mAbs) in cancer therapies is rapidly increasing. These therapies include general checkpoint inhibitors such as α-CTLA4 (cytotoxic T-lymphocyte-associated protein-4) or α-PD-1/PD-1L (programmed cell death-1 Ligand), and tumor specific antibodies [1,2,3]. To enhance antibody effector functions, various novel formats have been developed [4], including formats such as bispecific antibodies and mini/nanobodies that can be formulated to trigger novel effector functions [5]. Many of these studies aim to modify the natural effector functions of antibodies by improve binding and activity through Fc receptors such as the IgG Fc receptors (FcγR) [4]. Preferentially, this has to take place without introducing any immunogenic alterations nor altering the exceptionally long half-life of IgG1 [6]. 

Human and mouse FcγR are functionally and structurally conserved families of molecules. In mice, both the genetics and divergence are less complex than in humans. In general, both mice and humans have FcγRI, FcγRII, and FcγRIII receptors. In both species FcγRI is a very high nanomolar-affinity receptor, whereas the other FcγR have micromolar affinity. Mouse FcγRII (mFcγRII) and human FcγRIIb (hFcγRIIb) are orthologues and functional homologues, both containing Immunoreceptor Tyrosin-Inhibitory-Motif (ITIM), capable of down modulating Immunoreceptor Tyrosin-Activating Motifs (ITAM) found within the other activating FcγR or their associated γ-chain. Humans express the FcγRIIa which is the orthologue of mouse FcγRIII. These two receptors still differ fundamentally from each other, as human FcγRIIa uniquely contains cytoplasmic ITAM-motifs. Mice also have a receptor designated as FcγRIV [7], which is the orthologue of the human FcγRIII family. The human family of FcγRIII diverges into FcγRIIIa (found on some monocytes, macrophages, dendritic cells, and natural killer (NK) cells) and FcγRIIIb (found on granulocytes), with the former being a classical type-I transmembrane FcγR, while FcγRIIIb is a GPI-linked receptor found to reside primarily within lipid-rafts [8]. 

The Fc domain of mammalian IgG has a highly conserved N-linked glycosylation site in the second heavy chain constant domain (CH2) at the asparagine at position 297 (Asn297, EU numbering aligned to humans IgG) [9,10]. In both humans and mice this is a biantennary glycan, with variable composition [11]. In humans, most serum antibodies are fucosylated (around 94%), partially galactosylated and sialylated, and may contain a bisecting GlcNAc [11]. Afucosylated IgG appear to be generated specifically to cellular surface antigens, e.g., as provided by enveloped viral-glycoproteins and paternal antigens expressed on fetal cells [12,13,14]. Mouse IgG glycans are generally fully fucosylated and without bisecting GlcNAc [15]. Mice apply a different sialic acid capping being N-glycolylneuraminic (Neu5Gc) instead of N-acetylneuraminic acid (Neu5Ac) in humans. In addition, a α1-3-linked terminal galactose can be found on a minor fraction of mouse IgG which is not found in humans [15]. Of note, human glycans are not known to be immunogenic in mice as they are identical to those found in mice except for the sialylation, which is slightly different.

Modifying the Fc-glycan at position 297 greatly affects the interaction and function of the IgG [6]. For example, removing the glycan results in the inability of IgG to interact with both complement and FcγR. However, slight modifications, such as cleaving the sialic acids from the glycan of intravenous IgG (IVIg) has been reported to reduce its anti-inflammatory capacity in mice [16,17]. By contrast, removing the core-fucose results in a higher-affinity binding of IgG to human FcγRIIIa [12,18,19,20] and its orthologue in mice, FcγRIV [7,21,22,23]. The magnitude of enhanced affinity after afucosylation is ~20–40 [22,24] fold for human IgG1 binding to FcγRIIIa and 10 fold for mouse IgG2a binding to FcγRIV [21]. In addition, afucosylated IgG have no apparent effect on binding to the neonatal Fc receptor, responsible for the long half-life and placental transport of IgG [6], and as a consequence have unaltered half-life and placental transport in vivo [25,26]. Consequently, effector functions such as antibody dependent cellular cytotoxicity (ADCC) and phagocytosis (antibody dependent cell phagocytosis, ADCP) are increased [27]. Both the human FcγRIIIa/b [28] and mouse FcγRIV have a conserved N-linked glycan at position 162 that is responsible for discriminating between fucosylated and non-fucosylated IgG [22]. However, an increased affinity has also been reported for mouse FcγRII [21,22], but the presence of this effect and its magnitude remains to be confirmed. Taken together, these studies have shown that afucosylation of IgG is a viable option to enhance ADCC, with anti-CD20 therapies outperforming fucosylated variants in patients [29].

Novel mAb variants need to be tested in in vitro models, but also in in vivo models. Mice are one of the most popular experimental models to investigate cancer development and new potential therapies, partially because of their genetic homology with humans and our ability to easily (and relatively quickly) manipulate their genome. 

Human IgG1, the dominant subclass used for therapeutics in humans, has surprisingly similar affinity as mouse IgG2a to the murine receptor, including high- and medium affinity receptors FcγRI and FcγRIV [30]. In line with this, a potent immune activation with mouse effector cells was observed in vitro with human IgG1 [31]. Human IgG1 induced similar tumor cell lysis as mouse antibodies in vitro by mouse natural killer (NK) cells or polymorphonuclear cells (PMNs). Mouse macrophages were also able to kill tumor cells in the presence of any of the human IgG subclasses in an in vitro co-culture with the highest effect observed with IgG1. When macrophages were isolated from mFcγRI and mFcγRIII deficient mice, only mouse IgG2a, human IgG1, and human IgG3 were able to induce tumor cell death, suggesting that they act through the remaining activating Fcγ receptor, FcγRIV [31]. In addition, human IgG1 came in protective activity close to mouse IgG2a in in vivo models of anti-EGFR and anti-CD20 tumor mouse models [31]. 

In this study we investigated whether mice are a suitable model to test increased effector functions of human IgG1 (hIgG1) antibodies with lowered core fucosylation. To this end, we humanized the murine TA99 antibody targeting gp75 by replacing the constant regions of the murine IgG2a heavy chain for the human IgG1. With a co-culture of human effector cells and B16F10-gp75 tumor cells in the presence of the wildtype or modified antibody, we identified the primary human effector cells and confirmed its improved immune activation. We characterized binding affinities of hIgG1-TA99 as fucosylated and afucosylated variants on a biosensor array equipped with all mouse FcγR. Finally, we tested its capacity on tumor clearance of a B16F10-gp75 mouse melanoma in an intraperitoneal metastasis model. 

## 2. Materials and Methods

### 2.1. Antibodies

The development of the isotype control antibodies, anti-TNP, have been previously described [12,32]. The tumor targeting anti-gp75 antibodies (TA99) were generated using a similar process. The Variable Heavy and Light chain regions were designed with restriction overhangs and ordered from Geneart (Life Technologies, Paisley, UK). Subsequently, these regions were inserted in our expression vector for human IgG1 and the human kappa light chain [12,32,33].

For production, HEK-293F FreeStyle cell line expression system (Life Technologies) has been used. Antibody production was enhanced by co-transfecting vectors encoding p21, p27 and pSVLT as described in [34]. The afucosylated antibodies were generated by adding 400 µM 2-deoxy-2-fluoro-L-fucose (2F; Carbosynth, Compton, Berkshire, UK) during protein production [12].

All antibodies have been purified using Protein A HiTrapHP columns (GE Healthcare Life Sciences, Little Chalfront, UK) in the Akta-prime plus system (GE Healthcare Life Sciences) and dialyzed against PBS overnight. 

The level of fucosylation of the glucose at Asparagin257 was determined by proteolytically digesting the produced IgG with trypsin (Promega, Madison, WI, USA) and analyze the glycopeptide, which has virtually identical ionization levels with and without fucose by liquid chromatography-mass spectrometry (LC-MS) as previously described [35,36,37,38].

Antibodies used for flow cytometry are anti-human CD16 (CD16, eBioscience, San Diego, CA, USA), CD32 (6c4, eBioscience), CD64 (10.1, eBioscience), and HLA (L243, Biolegend, San Diego, CA, USA), and anti-mouse CD3e (145-2c11, eBioscience), F4/80 (BM8, eBioscience), GR1 (RB6-8C5, eBioscience), NK1,1/CD161 (PK136, eBioscience), and NKp46/CD335 (29A1.4, eBioscience).

### 2.2. Isolation of Primary Effector Cells

Human effector cells were isolated from peripheral blood obtained from healthy donors or buffycoats from Sanquin blood supply (Amsterdam, The Netherlands). To obtain PMNs, Lymphoprep (Axis-Shield, Oslo, Norway), density gradient centrifugation was used to separate the blood. Erythrocytes, present together with the PMNs in the dense fraction, were lysed in ammonium chloride buffer (155 mM NH_4_Cl, 10 mM KHCO_3_, and 0.1 mM EDTA). The remaining cell population, the PMNs, were washed with PBS (B.Braun, Melsungen, Germany), resuspended in complete medium, and left to rest at 37 °C prior to the experiment. The mononuclear layer (Peripheral blood mononuclear cells, PBMC), containing a.o. the NK cells and C14^+^ monocytes, obtained from the Lymphoprep separation was washed with PBS and resuspended in complete medium. To obtain NK cells and CD14^+^ monocytes specifically, cell separation beads (Miltenyi Biotech, Leiden, The Netherlands) were used according to manufacturer’s protocol, washed with PBS, and resuspended in complete medium. Peripheral blood lymphocytes (PBLs) were obtained as flow through from the CD14^+^ monocyte isolation, washed, and resuspended in complete medium.

### 2.3. Cell Culture

The B16F10-gp75 mouse melanoma cell line was generated to stably express gp75 on the cell membrane as described in [39]. B16F10-gp75 tumor cells were cultured under humidified conditions (37 °C, 5% CO_2_) in medium RPMI 1640 (Gibco, Paisley, UK) supplemented with 10% heat inactivated foetal calf serum (FCS, Lonza, Verviers, Belgium), glutamine (Glutamax, Lonza), and penicillin/streptavidin (Lonza), hereafter referred to as ‘complete medium’. 

Macrophages were generated from the CD14^+^ monocytes isolated from blood as described above. Monocytes were cultured for 6 days in the presence of 50 ng/mL macrophage-colony stimulating factor (M-CSF) (eBioscience, San Diego, CA, USA) in complete medium. At day 6, cells were harvested and seeded in multi well plates for the experiment, after which they were cultured for another two days in complete medium with 50 ng/mL M-CSF. 

### 2.4. Flow Cytometry

The expression of IgG Fc receptors (FcγR) was determined using flow cytometry. Samples were blocked with 5% serum, of the same species (human or mouse) as the cells, diluted in PBS with 0.5% bovine serum albumin (BSA). Fluorescently labelled antibodies targeting the Fcγ receptors were diluted in 0.5% PBS/BSA, added to the cells, and incubated on ice. For the mouse samples, cells were washed with 0.5% PBS/BSA and erythrocytes were lysed with lysing solution diluted in MilliQ (10× concentrate, BD Biosciences, Franklin Lakes, NJ, USA). Data was acquired with BD LSRFortessa X-20 (BD Biosciences) and analyzed with Flowjo X (Flowjo, LLC, Ashland, OR, USA). 

### 2.5. Antibody Dependent Cellular Cytotoxicity (ADCC)

The target tumor cells B16F10-gp75 were seeded at a concentration of 8000 cells/well in a 96 wells plate. Effector cells were isolated as described above and prepared in complete medium. The effector to target (E:T) ratio for PMNs was 80:1, for PBMCs, PBLs, and monocytes 10:1 and for NK cells 5:1. Antibodies were diluted to 1 µg/mL or 5 µg/mL in complete medium. To analyze target cell death, the co-culture plates were carefully washed after 4 h of incubation to remove effector cells. After washing, CellTiterBlue (Promega, Leiden, The Netherlands) was added and co-incubated for three hours, according to manufactures protocol to stain remaining viable target cells. As no proliferation of target or effector cells is expected within 4 h no increase was expected, we interpret loss of CellTiterBlue signal to be a loss of viable cells. Readout was performed on a Bio-rad Model 680 Microplate Reader (Bio-rad, Hercules, CA, USA) and expressed as %remaining cell (compared to no antibody-treated cells). 

### 2.6. Antibody Dependent Phagocytosis (ADCP)

Antibody dependent phagocytosis was performed as described in [40]. In short, macrophages were seeded in the presence of M-CSF for 2 days prior to the experiment in 24 wells plates (200,000/well). At day 0 B16F10-gp75 were harvested and stained with cell proliferation dye eFluor 450 (eBioscience) according to manufacturer’s protocol. Both antibodies (1 µg/mL) and tumor cells (E:T = 15:1) were diluted in complete medium. After 24 h of co-culture at 37 °C, cells were collected with Trypsin/EDTA and scratching. Subsequently samples were blocked with human serum, stained with anti-HLA-DR to stain macrophages, and fixed with 4% paraformaldehyde in PBS. Data was acquired and analyzed with the BD LSRFortessa X-20 and Flowjo X. The percentage of double-positive (HLA-DR^+^, eFluor 450^+^) macrophages was determined. 

### 2.7. B16 Mouse Melanoma Metastasis Model

8-week-old C57Bl/6 mice were obtained from Harlan. 8–10 week old C57Bl/6J FcγRIV^−/−^ were generated [41]. Mice were kept under standard conditions in the university animal facilities of the VUmc and LUMC with unrestricted access to food and water and wellbeing was observed on daily basis. At the morning of the experiment, B16F10-gp75 tumor cells were harvested and washed in PBS. The cells were resuspended in PBS at 50,000 cells/300 µL. The tumor targeting or isotype antibodies were diluted to 50 µg/300 µL in PBS. Mice were intraperitoneally injected with first tumor cells right of the central line followed by antibodies left of the central line. 14 days post injection, mice were sacrificed with CO_2_ and peritoneal metastasis were scored. 

### 2.8. Surface Plasmon Resonance

Affinity of all IgG-FcγR was performed as previously [30]. In short, biotinylated mouse FcγRI, FcγRII, and FcγRIV Sino Biologicals (Bejing, China) were used. Biotinylated mouse FcγRIII was not available. They were spotted using a Continuous Flow Microspotter (Wasatch Microfluidics, Salt Lake City, UT, USA) on a SensEye G-streptavidin sensor array (Senss, Enschede, Netherlands). Biotinylated Fcγ receptors were spotted in duplo in three-fold dilutions, ranging from 100 nM to 3 nM for FcγRII and 30 nM to 1 nM for FcγRI and FcγRIV in PBS 0.075% Tween-80 pH 7.4 (Amresco, Solon, OH, USA). Biotinylated anti-His IgG1 (GenScript, Piscataway, NJ, USA) was spotted in duplo and three-fold dilution, ranging from 100 nM to 3 nM, to capture 30 nM His-tagged FcγRIII (Sino Biologicals), equally diluted in PBS 0.075% Tween-80, pH 7.4) which was loaded onto the sensor before every antibody injection in the IBIS MX96 (IBIS technologies, Enschede, The Netherlands) SPR unit. Antibodies were then injected over SPR chip at 1.5 dilutions from 3.9 nM to 337.5 nM in PBS in 0.075% Tween-80. Regeneration with acid buffer (100 nM H_3_PO_4_, 0.075% Tween 80, pH 1.5) was carried out after every sample. The dissociation constant (K_D_) was calculated for each ligand concentration by equilibrium fitting, and reported at by interpolation Rmax = 500 as described in [30]. For this hist-tagged FcγRIII, anti-His association and dissociation curves were subtracted before calculation of IgG-binding affinity using SPRINT 1.9.4.4 software (IBIS technologies). All binding data was analyzed using Scrubber software version 2 (Biologic Software, Campbell, Australia).

### 2.9. Statistical Analysis

GraphPad Prism 6 was used for data analysis. Data depicted are mean ± SEM. Data was analyzed with a 1way ANOVA followed by Tukey’s multiple comparison test.

## 3. Results

### 3.1. Lack of Core-Fucose in Human IgG1 Increases the Activation of Human Effector Cells

**Figure 1:**
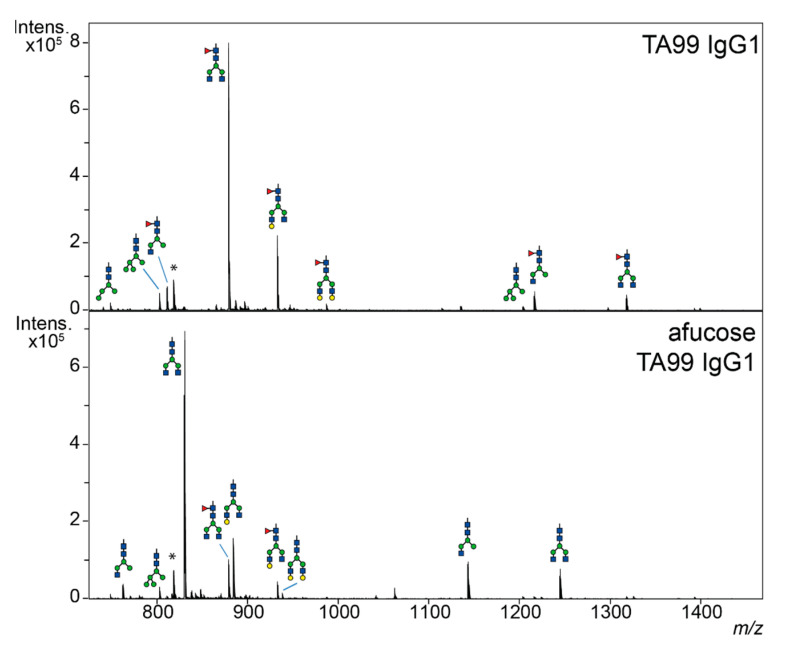
Afucosylated antibodies have minimal expression of fucose on glycans. LC-MS spectra of trypsin-generated human IgG1 glycopeptides. Glycopeptide species were observed in both triple-charged form (below *m*/*z* 1000) and double charged form (above *m*/*z* 1000). Top humanized fucosylated hIgG1-TA99 and bottom, afucosylated hIgG1-TA99. Green circle = mannose; yellow circle = galactose; blue square = *N*-acetylglucosamine; red triangle = fucose; * = unidentified peptide.

**Figure 2:**
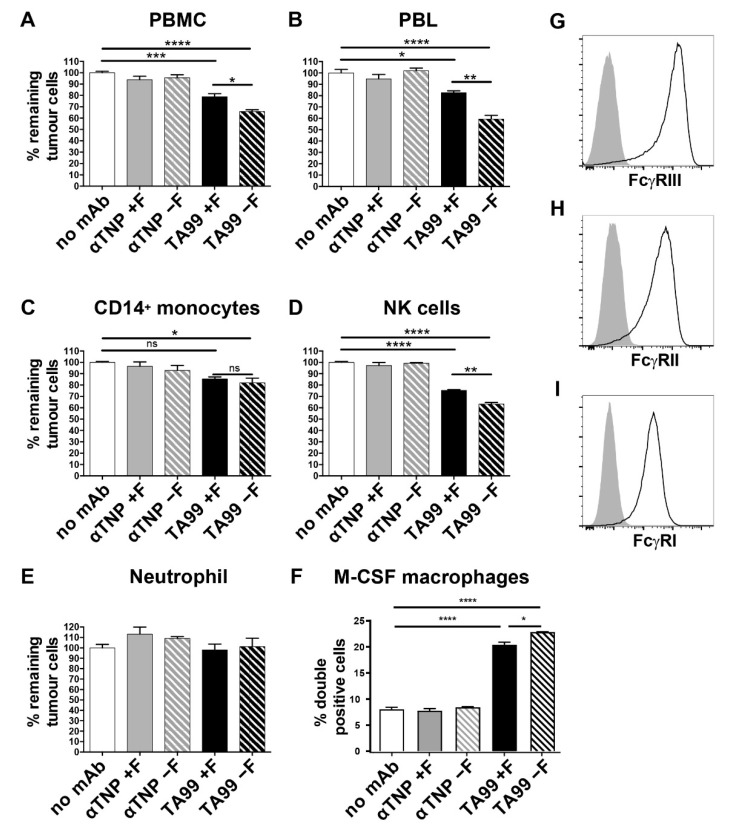
Lack of core fucose in the IgG1 Fc domain enhances antibody-mediated tumor killing by natural killer cells and human macrophages. (**A**–**E**) ADCC or ADCP (**F**) with B16F10-gp75 cells as targets opsonized with anti-GP75 TA99 hIgG1 (black bars) or control (grey bars, anti-2,4,6-trinitrophenol, TNP) antibodies, with or without core fucose (+F solid and -F striped bars resp.) by: (**A**) PBMC (**D**) PBL, (**C**) CD14^+^ monocytes, (**D**) NK cells or (**E**) PMN cells (predominantly neutrophils). % Remaining tumor cells relative to the no antibody (white bars) co-culture were used as readout (**F**) ADCP by M-CSF cultured CD14^+^ monocyte derived macrophages. Percentage of tumor cell^+^—macrophages in the co-culture. (**G**–**I**) FcγRIII (CD16) (**H**) FcγRII (CD32) (**I**) FcγRI (CD64) expression on M-CSF cultured CD14^+^ monocyte-derived macrophages was determined by flow cytometry. Plots and graphs represent data obtained in 3 to 5 independent experiments and healthy donors. All graphs represent mean ±SEM. * *p* ≤ 0.05; ** *p* ≤ 0.01; *** *p* ≤ 0.001; **** *p* < 0.0001, ns = Not Significant *p* > 0.05.

First, humanized tumor-targeting anti-gp75 antibodies (hIgG1-TA99) were produced in HEK cells without and with 2-deoxy-fluorofucose generating fucosylated and afucosylated IgG, respectively, and purified by affinity chromatography. Their glycosylation profiles were then investigated by LC-MS of tryptic glycopeptides encompassing the 297-N-linked glycan. Wildtype hIgG1-TA99 was highly fucosylated, whilst fucosylation was found to be strongly reduced in our hypo-/afucosylated hIgG1-TA99 variant (Figure 1). B16F10-gp75 mouse melanoma cells were then co-cultured with various human effector cells in the presence of humanized tumor targeting anti-gp75 antibodies (hIgG1-TA99) or nonspecific antibodies in antibody dependent tumor killing assays. Antibody-mediated killing was monitored using the CellTiter-Blue viability assay as proxy for ADCC. Peripheral blood mononuclear cells (PBMC; which contain NK cells and monocytes as effector cells) induced B16F10-gp75 tumor cell killing in the presence of hIgG1-TA99, which was significantly increased with the afucosylated variant (Figure 2A). Peripheral blood lymphocytes (PBL, containing NK cells, but not monocytes) induced similar ADCC as PMBC, again with the afucosylated IgG1-TA99 being more potent (Figure 2B). Isolated CD14^+^ monocytes hardly induced ADCC towards B16F10-gp75 cells in the presence of either fucosylated or fucosylated hIgG1-TA99 antibodies (Figure 2C), whereas isolated NK cells were very effective (Figure 2D), supporting that NK cells were the primary effector cells within the PBMC population. Neutrophils, the most abundant cytotoxic cell population in blood, did not show any capacity to kill tumor cells even with high E:T ratios and increased antibody concentrations (Figure 2E). CD14^+^ monocyte derived M-CSF macrophages efficiently induced ADCP of B16F10-gp75 cells, particularly through afucosylated hIgG1-TA99, which resulted in a significantly decreased number of remaining tumor cells and an increased number of tumor-cell positive macrophages, which we previously showed to reflect phagocytosis (Figure 2F and [40]). M-CSF-cultured macrophages expressed FcγRI, FcγRII, and FcγRIII (Figure 2G–I).

Several studies have demonstrated that low core-fucosylation increases the interaction of human IgG1 to human FcγRIIIa and FcγRIIIb (CD16) [12,28,37,42,43,44]. Human neutrophils express FcγRIIIb, whereas NK cells, and a small population of peripheral monocytes (CD14^+^), express FcγRIIIa [45,46,47,48]. These data support that CD16a^+^ human NK cells and macrophages are important effector cells due to their expression of the transmembrane FcγRIIIa form, while neutrophils express the glycosylphosphatidyl-inositol (GPI)-linked FcγRIIIb glycoforms that does not induce ADCC.

### 3.2. Mice Treated with Afucosylated hIgG1-TA99 Develop Less Peritoneal Metastasis

**Figure 3:**
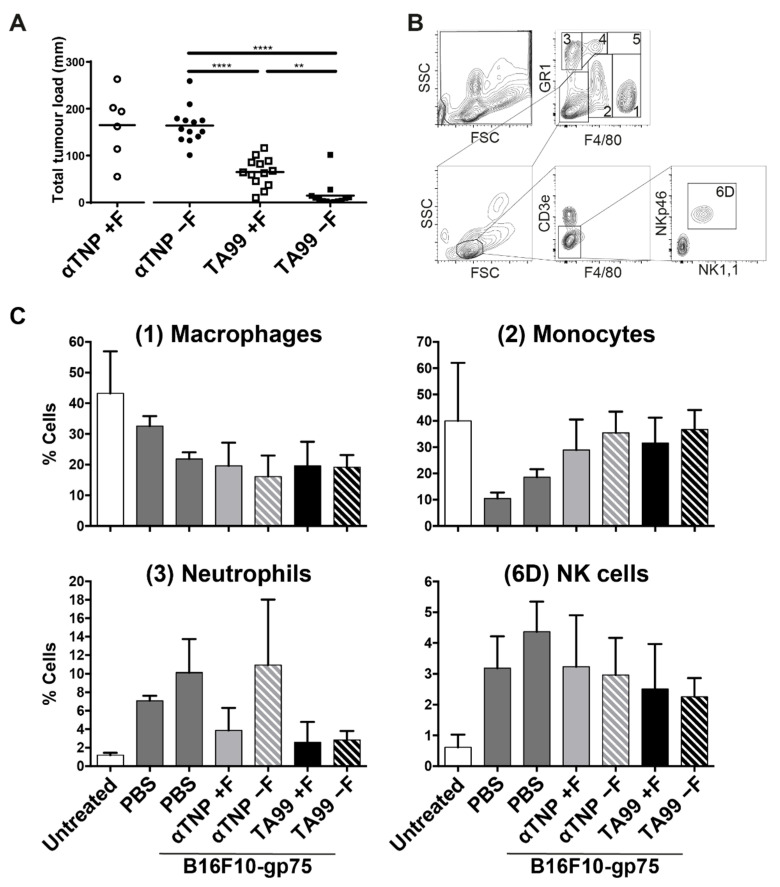
Treatment with afucosylated jIgG1-TA99 decreased tumor outgrowth in vivo. (**A**) C57Bl/6 mice were injected intraperitoneally with 50.000 B16F10-gp75 and 50 μg nonspecific (○/●) or tumor specific hIgG1 TA199 antibodies) that were either fucosylated (□) or hypo-fucosylated (■). Fourteen days post injection mice were sacrificed and metastasis outgrowth in the peritoneum was scored. N = 6 for αTNP +Fucose, *n* = 13 for the other groups. (**B**) Populations identified in a peritoneal lavage. 5 populations are gated in a F4/80/GR1 plot, (1) F4/80^+^GR1^−^, (2) F4/80^int^GR1^int^, (3) F4/80^−^GR1^+^, (4) F4/80^int^GR1^+^, (5) F4/80^+^GR1^+^. The negative population was used to gate lymphocytes, CD3^−^ cells and 6D) NK cells respectively. (**C**) Composition of the dominant myeloid and NK—effector populations in a peritoneal lavage of mice 24 h after intraperitoneal injection with PBS, B16F10-gp75 with or without antibodies. *N* ≥ 4. ** *p* ≤ 0.01, **** *p* ≤ 0.0001.

Previous studies have shown mixed results using afucosylated antibodies in mice, most showing a beneficial effect [21,49,50,51], although some have shown no effect of IgG-afucosylation in a subcutaneous tumor model [51], or shown no effect of afucosylated trastuzumab in wildtype-mice [50,52], but only in hFcγRIIIa-transgenic mice [50]. In those studies showing the beneficial effect, the contributing receptor was not identified. We investigated if afucosylated therapeutic IgG is beneficial in a peritoneal metastasis B16F10-gp75 model. High tumor outgrowth was observed in mice that had been treated with nonspecific humanized fucosylated or afucosylated anti-TNP antibodies (Figure 3A). A significant reduction of tumor outgrowth was found in mice treated with hIgG1-TA99 compared to the nonspecific antibodies. Mice treated with afucosylated hIgG1-TA99 showed almost no tumor outgrowth, except for a few individual mice (Figure 3A).

In the peritoneal lavage of untreated mice, the vast majority of the effector cells were F4/80 expressing cells (macrophages, F4/80^+^GR1^−^ and monocytes, F4/80^int^), followed by neutrophils (GR1^+^) and NK cells (CD3^−^NKp46^+^NK1,1^+^) (Figure 3B,C). When PBS was injected in the peritoneal cavity, an influx of NK cells and neutrophils in the first 24 h was observed (Figure 3C). 

### 3.3. Afucosylated Human IgG1 Has Increased Affinity for Mouse FcγRIV 

**Figure 4:**
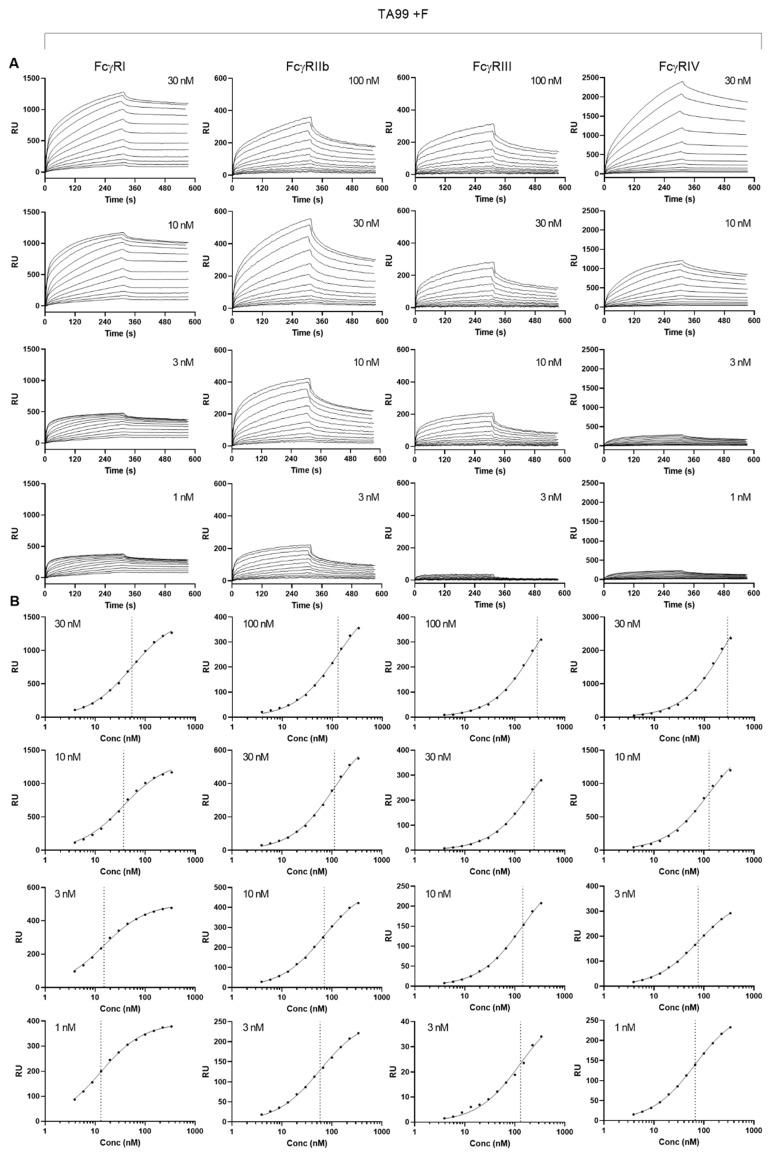
Affinity plots for fucosylated human IgG1 to mouse FcγR. Binding affinity was determined by Surface plasmon resonance using C-terminally site-specifically biotinylated FcγR coupled to streptavidin sensor arrays. hIgG1-TA99 flowed over the chip at concentrations ranging from 3.9 nM until to 337.5 nM at 1.5 dilutions for the different mouse FcγR at different densities as indicated. (**A**) Sensorgrams and (**B**) derived affinity plots. The affinities found at different receptor densities in (**B**) are indicated by vertical dotted lines, and used to calculated and interpolated of KD for Rmax of 500 (Figure 5).

**Figure 5:**
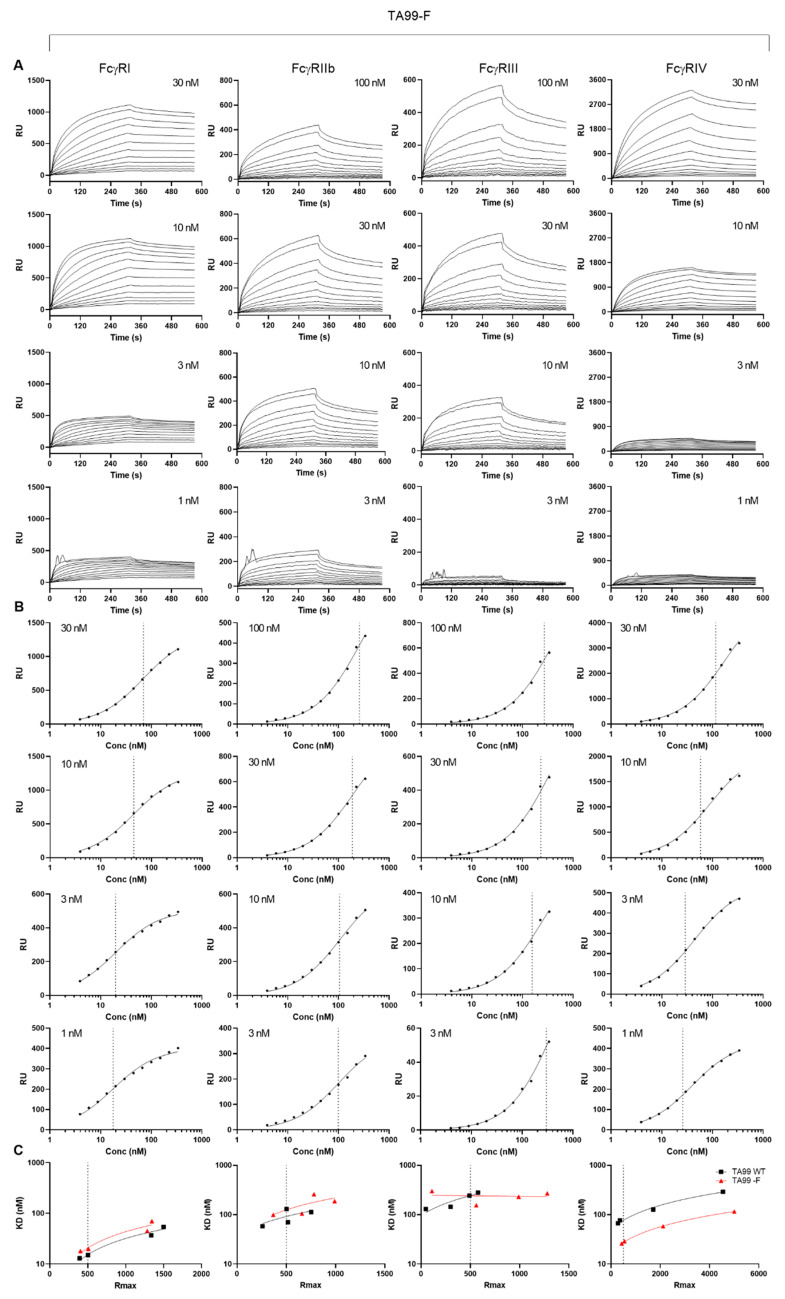
Affinity plots for afucosylated human IgG1 to mouse FcγR: Lack of fucose in the Fc domain results in increased binding to mouse FcγRIV. Experiment was carried out as described for Figure 4, with (**A**) Sensorgrams and (**B**) derived affinity plots for 4 different ligand (FcγR) concentrations as indicated. (**C**) The derived KD from each affinity plots for fucosylated hIgG1 (Figure 4B) and afucosylated IgG1 from (**B**) and Rmax of each ligand concentration plotted for interpolation of KD for both fucosylated and afucosylated IgG1 to a constant Rmax of 500 (vertical lines, tabulated in Table 1).

**Figure 6:**
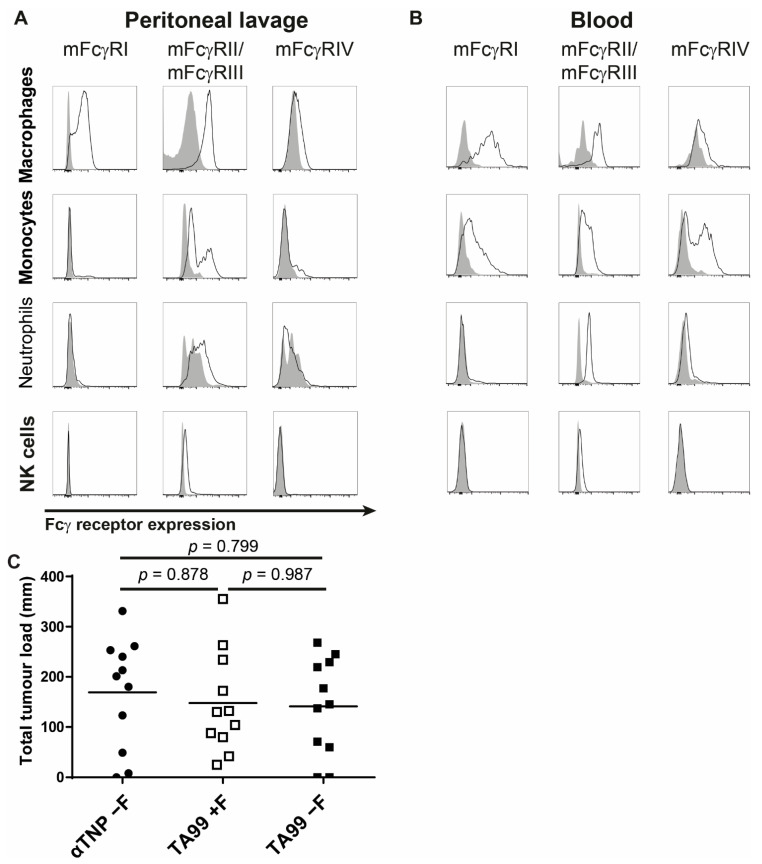
FcγRIV is essential in antibody therapy in the peritoneal cavity. (**A**,**B**) Fcγ receptor expression by effector cells in the peritoneal cavity (**A**) and in blood (**B**). Isotype control is shown in grey, FcγR-specific antibodies with a solid line. (**C**) C57Bl/6 mice lacking FcγRIV were injected intraperitoneally with 50,000 B16F10-gp75 and 50 μg nonspecific hypo-fucosylated hIgG1-anti-TNP (●) or humanized IgG1-TA99as either fucosylated (□) or hypo-fucosylated (■) variant. 14 Days post injection mice were sacrificed and metastasis outgrowth in the peritoneum was scored. *N* = 11 per group.

We then investigated the apparent affinities of normally fucosylated and afucosylated hIgG1-TA99 using a plasmon surface resonance array equipped with all mouse FcγR [30], enabling us to measure binding of a single IgG to all receptors simultaneously. Although the fast on-rates and possibly two-phase dissociation may suggest some presence of aggregates, this was very similar for both fucosylated and afucosylated IgG. Generally, the binding behavior of both antibodies were similar. To compare general affinity differences, we chose to use a simplified 1:1 Langmuir model that does not fully represent the actual interaction which is more complicated (Figure 4 and Figure 5) [30]. In addition, while comparing afucosylated IgG with fucosylated IgG, we considered meaningful differences to be higher than 2 as this is in the sensitivity range of SPR. We found that afucosylation of human IgG1 did not, or hardly, affect binding to FcγRI, FcγRII, and FcγRIII (<2 fold, Figure 5 and Figure 6 and Table 1), whereas the binding to FcγRIV was increased three-fold (Figure 5, Table 1). 

### 3.4. Interaction of Afucosylated Human IgG1 with FcγRIV Is Crucial for Elevated Tumor Clearance 

To explain the increased in vivo efficacy of afucosylated hIgG1-TA99 (Figure 3A) we first analyzed FcγR expression of the different effector populations in the peritoneal cavity and blood. Peritoneal macrophages (F4/80^+^) and monocytes (F4/80^int^) showed low expression of FcγRIV whereas no expression was observed on neutrophils (GR1^+^) or NK cells (CD3^−^NKp46^+^NK1,1^+^) (Figure 6A). Populations of F4/80 high and low-expressing cells were identified in blood that differed in FcγR-profiles. Both expressed high FcγRI and FcγRIV (Figure 6B). We then tested if FcγRIV is responsible for the beneficial effect of treating mice with afucosylated IgG1-TA99. Mice with a genetic deletion in FcγRIV (FcγRIV^−/−^) showed that therapeutic ability of hIgG1-TA99 in the B16F10-gp75 melanoma model was abolished with no further enhancement of afucosylation. (Figure 6C). This indicates that FcγRIV was the primary or even the sole receptor involved in the tumor clearance, and responsible for mediating enhanced anti-tumor immunity when treating with afucosylated anti-tumor human IgG1.

## 4. Discussion

Antibodies are rapidly gaining ground as cancer therapeutics due to continuous and rapid discovery of new targets and ways to improve antibody effector functions. One promising modification strategy is changing glycosylation of mAbs, as this can enhance binding to human FcγRIIIa. This mimics a natural variation seen in antibodies that seem to form specifically to membrane embedded proteins in host cells, such as that of enveloped viruses [14], potentially providing stronger protection through enhanced FcγRIIIa-mediated effector functions as seen in elite-controller of HIV infections [53]. However, in some infections such as dengue fever [54] and COVID-19, this seem to cause more harm than good due to immune overactivation and inflammatory responses [14]. Currently, various afucosylated antibodies are in clinical trials and a few are also approved for the treatment of hematological cancer types [55,56]. Here we tested if mice represent a suitable model organism to investigate afucosylated mAbs and provide a formal proof that enhanced therapeutic effect of such glycoengineered IgG is because of enhanced binding to mouse FcγRIV.

Several potential caveats are already known for addressing this question in mice. The expression pattern of the only FcγR capable of sensing between fucosylated and non-fucosylated IgG, the orthologue hFcγRIIIa (and hFcγRIIIb) and the orthologue mFcγRIV, differs somewhat between the species [57]. Mouse monocytes, macrophages and neutrophils should theoretically be activated preferentially by non-fucosylated IgG as these cells have been reported express FcγRIV [57]. hFcγRIIIa, which is functionally most similar to mFcγRIV, is also expressed on monocytes/macrophages in humans but not on granulocytes, while it is expressed on human NK cells [57]. Human neutrophils express FcγRIIIb that is capable of enhanced phagocytosis through afucosylated IgG but not ADCC through this GPI-linked receptor [58,59,60]. Thus, taken together, monocytes/macrophage population in both species, but only human NK cell and mouse neutrophils, have built in ADCC-enhancing mechanism against targets opsonized with afucosylated-IgG through hFcγRIIIa and mFcγRIV. 

In vitro only human NK cells and monocyte derived M-CSF macrophages are involved in ADCC or ADCP of mouse B16F10-gp75 melanoma cells in the presence of humanized hIgG1-TA99 [61,62]. The target, gp75, is normally not expressed on the surface, by B16F10 cells in vitro, yet targeting antibodies are protective in vivo. Providing gp75 variant that does induce surface expression allows also in vitro targeting [39]. While elevated antigen density enhances the target cell sensitivity to ADCC, IgG-afucosylation can boost ADCC their potential for targets with low expression [63,64,65]. Indeed, without Fc core fucosylation these antibodies provided a significantly enhanced killing, presumably through its enhanced binding to FcγRIIIa, which is expressed by both NK cells and macrophages. Afucosylation also provides enhanced binding to FcγRIIIb on neutrophils, but these cells were unable to induce killing irrespective of fucosylation status of these antibodies. Although neutrophils have been found to be able to kill some tumor targets, this killing seems not to proceed through FcγRIIIb, but rather via FcγRIIa [60]. Enhanced targeting to FcγRIIIb by afucosylation of the anti-tumor IgG has even been found to adversely affect tumor-killing potential by neutrophils, presumably by redirecting the effector functions away from the ITAM-linked FcγRIIa to the GPI-linked FcγRIIIb [59,60]. 

Human IgG1 and mouse IgG2a share similar strength of effector functions [30], and human IgG1 has been shown to provide strong mouse effector cells in in vitro tumor models [31]. We observed significantly better prevention of metastasis outgrowth in mice treated with afucosylated hIgG1-TA99 compared with wildtype hhIgG1-TA99. The effect of core fucosylation in the Fc domain of human IgG1 influences binding to both orthologue receptors, human FcγRIIIa [20] and mouse FcγRIV [21]. This was confirmed by analyzing the binding capacity between highly fucosylated (wildtype) and afucosylated hhIgG1-TA99, which showed only an influence on binding to mFcγRIV. This is in line with previous studies. In humans and mice, afucosylation of IgG most strongly affects binding to hFcγRIIIa/b [37,66,67,68] and mFcγRIV [21,22]. However, the literature is not unanimous when it comes to effects on the inhibitory FcγRIIb, which has also been shown to have a light preference for afucosylated IgG in some reports [21,67,68], but not all [37] in both species. The reported increase in FcγRIIb binding also seems subclass specific, with mIgG2a and mIgG2b possibly being affected, but not mIgG1 [21,22]. Most reports however agree that the effect on FcγRIIb binding (~2 fold) is far less than for mFcγRIV, with the notable exception of one report showing similar increased binding for both mFcγRIIb and mFcγRIV [21]. Here we found only mFcγRIV binding to be affected by afucosylation of human IgG1, and therefore the afucosylation should tilt responses away from inhibitory FcγRIIb toward activating responses in mice. The enhanced binding was modest, i.e., only three times enhanced affinity, yet it did translate into meaningful differences in vivo.

Similar to the enhanced in vitro ADCC by human effector cells using afucosylated hIgG1-TA99, we found that these antibodies also provided better protection in an intraperitoneal tumor model in mice with B16F10-gp75 cells. This is in line with previous studies using either human or mouse afucosylated antibodies in various tumor models [21,49,50,51,52]. None of these studies identified the receptor involved in the enhanced therapy with afucosylated IgG. Here we identified FcγRIV to be responsible for both the therapeutic effect in general as well as the additional gain of treating with afucosylated hIgG1-TA99, which is in agreement with the enhanced binding of afucosylated hIgG1 to only FcγRIV. This indicates that the interactions between hIgG1 and human FcγRIIIa or its orthologue mouse FcγRIV are conserved between these species, and that the mouse can be a suitable model organism to further optimize antibodies for clinical use.

Although both human and mouse macrophages express the FcγR involved in improved killing induced by afucosylated mAbs, mouse models have some limitations. Mice and humans differ in the potential additional effects of NK cells and neutrophils in recruiting a beneficial effect by afucosylation of IgG. In humans, NK cells express FcγRIIIa, enabling enhance ADCC by afucosylated IgG, with potential negative effect by FcγRIIIb by PMN [59]. Mouse NK cells, however, do not express FcγRIV, but mouse PMN can induce its expression, for example after thioglycolate injection [7,57]. Mouse NK cells only express the low affinity receptor FcγRIII [39,48,61,69,70,71]. Interestingly, although neutrophils have been shown to be crucial in tumor rejection in a subcutaneous mouse model using TA99 antibodies [72], this does not seem to be the case in the intraperitoneal metastasis model where macrophages seem to be the dominant effector cells [73,74,75,76,77,78]. Overall, these data suggest FcγR to expression patterns to have evolved differently between human and mouse myeloid- and NK cells, and underline the redundant functionality of the FcγR family. Our data further support that the main effector mechanism in mice is ADCP/ADCC by FcγRIV-expressing macrophages, and not via ADCC by NK cells. In the end, it is likely that the effector phase of these receptors may largely depend on the cellular context and the cellular machinery expressed and equipped by each effector cell. 

Here, we observed an influx of primarily neutrophils, monocytes, and NK cells after injection with B16F10-gp75 tumor cells into the peritoneal cavity. Therapeutic application of these antibodies did not significantly alter influx of these cells in to the peritoneum. Of the cells present in the peritoneal cavity, only peritoneal macrophages and monocytes expressed FcγRIV. Since we observed no suppression of tumor outgrowth in FcγRIV^−/−^ mice in the presence of tumor targeting mAbs, our data support that monocytes and macrophages were the primary immune cells in the peritoneal cavity of mice. Even though other FcγR expressing cells such as NK cells and neutrophils were present and easily recruited, they likely did not significantly contribute to the cytotoxic anti-tumor responses, due to the absence of FcγRIV expression. 

It was previously reported that the primary FcγR to mediate effective mAb therapy differs between distinct locations of metastasis [21,79,80]. Otten et al. demonstrated that the high affinity receptors FcγRI and IV have redundant functions in a liver metastasis model. Knocking out or blocking one of the two was not sufficient to abolish antibody treatment effects on metastasis outgrowth. Only when both receptors were absent was mAb therapy unsuccessful in preventing development of liver metastasis [80]. Similar to the liver, both the FcγRI and FcγRIV have been described in two independent papers as the primary receptor for antibody therapy in the lungs. Nimmerjahn and Ravetch demonstrated that FcγRIV was responsible for therapeutic effect in a B16F10 lung metastasis model [21]. On the contrary, Bevaart et al. identified FcγRI as the main FcγR for mAb therapy in the B16F10 lung model [79]. The discrepancy in the outcomes in these latter two studies has not been clarified. Nonetheless, it is likely that tissue specific cell types are responsible for local effects of therapeutic mAbs, which might also be the case in the peritoneal cavity where, for example, in the omentum colonies of macrophages have been found that might be involved in this process [81]. 

## 5. Conclusions

Our data demonstrate that afucosylated human IgG1 induces increased tumor killing by human NK cells and macrophages via binding to FcγRIIIa. Importantly, afucosylated hIgG1 antibodies are also preferentially recognized by its orthologue receptor in mice, FcγRIV, on monocytes and/or macrophages. The potential of afucosylated human IgG1 antibodies can therefore be tested in therapeutic mouse models, which may simplify the road towards preclinical screening of candidate antibodies and the mechanisms of antibody-mediated tumor clearance.

## Figures and Tables

**Table 1:** Dissociation constant (Kd) of humanized IgG1-TA99 mAbs and mouse Fcγ receptors.

	FcγRI	FcγRII	FcγRIII	FcγRIV
TA99 hIgG1 wt	1.34 × 10^−9^	0.93 × 10^−7^	2.24 × 10^−7^	8.97 × 10^−8^
TA99 hIgG1 low fuc	1.6 × 10^−9^	1.35 × 10^−7^	1.90 × 10^−7^	2.95 × 10^−8^
Fold change	0.80	0.69	1.18	3.04

## Data Availability

All data are contained within the article.
